# The Future of Gene Therapy for Transfusion-Dependent Beta-Thalassemia: The Power of the Lentiviral Vector for Genetically Modified Hematopoietic Stem Cells

**DOI:** 10.3389/fphar.2021.730873

**Published:** 2021-10-01

**Authors:** Parin Rattananon, Usanarat Anurathapan, Kanit Bhukhai, Suradej Hongeng

**Affiliations:** ^1^ Department of Pediatrics, Faculty of Medicine Ramathibodi Hospital, Mahidol University, Ratchathewi, Thailand; ^2^ Department of Physiology, Faculty of Science, Mahidol University, Ratchathewi, Thailand

**Keywords:** β-thalassemia, hematopoietic stem cells (HSCs), gene therapy, lentiviral vector, transfusion-dependent, allogeneic HSC transplantation

## Abstract

β-thalassemia, a disease that results from defects in β-globin synthesis, leads to an imbalance of β- and α-globin chains and an excess of α chains. Defective erythroid maturation, ineffective erythropoiesis, and shortened red blood cell survival are commonly observed in most β-thalassemia patients. In severe cases, blood transfusion is considered as a mainstay therapy; however, regular blood transfusions result in chronic iron overload with life-threatening complications, e.g., endocrine dysfunction, cardiomyopathy, liver disease, and ultimately premature death. Therefore, transplantation of healthy hematopoietic stem cells (HSCs) is considered an alternative treatment. Patients with a compatible human leukocyte antigen (HLA) matched donor can be cured by allogeneic HSC transplantation. However, some recipients faced a high risk of morbidity/mortality due to graft versus host disease or graft failure, while a majority of patients do not have such HLA match-related donors. Currently, the infusion of autologous HSCs modified with a lentiviral vector expressing the β-globin gene into the erythroid progenitors of the patient is a promising approach to completely cure β-thalassemia. Here, we discuss a history of β-thalassemia treatments and limitations, in particular the development of β-globin lentiviral vectors, with emphasis on clinical applications and future perspectives in a new era of medicine.

## Introduction

β-thalassemia belongs to a family of inherited hemoglobin disorders and is characterized by a quantitative reduction in β-globin chains. β-thalassemia has a wide range of clinical severity, from severe transfusion-dependent thalassemia major to the highly variable non-transfusion-dependent thalassemia intermedia. More than 200 mutations in the β-globin gene have been reported (Olivieri, 1999; Giardine et al., 2007). The mutations include the following: mutations affecting transcription, RNA processing, or RNA translation; small insertions or deletions within the gene; single base substitutions; mutations affecting translation initiation, elongation, termination, and more rarely, deletions of a substantial proportion of the regulatory elements in the locus control region (LCR) or deletions of the open reading frame (Thein, 1998), resulting in either a complete absence (β^0^-thalassemia), or a partial deficiency (β+-thalassemia) of β chains.

In normal individuals, there is a balance between α- and β-globin chain synthesis. In individuals with β-thalassemia, mutations lead to imbalanced globin chain synthesis and an excess of α chains. Unbound α-globin chains precipitate in red blood cell precursors and their progeny causing cellular damage, a process that leads to defective erythroid maturation, ineffective erythropoiesis, and shortened red blood cell (RBC) survival (Weatherall, 1998). Ineffective erythropoiesis combined with shortened RBC survival leads to anemia. If left untreated, the disease could induce an expansion of marrow cavities and massive extramedullary cell proliferation resulting in skeletal deformities, hepatosplenomegaly, and extramedullary pseudotumors (Haidar et al., 2010). Erythroid hyperplasia and ineffective erythropoiesis are responsible for increased intestine iron absorption, which, in addition to regular blood transfusion, results in chronic iron overload with life-threatening complications such as endocrine dysfunction, cardiomyopathy, liver disease, and ultimately, premature death.

**FIGURE 1:**
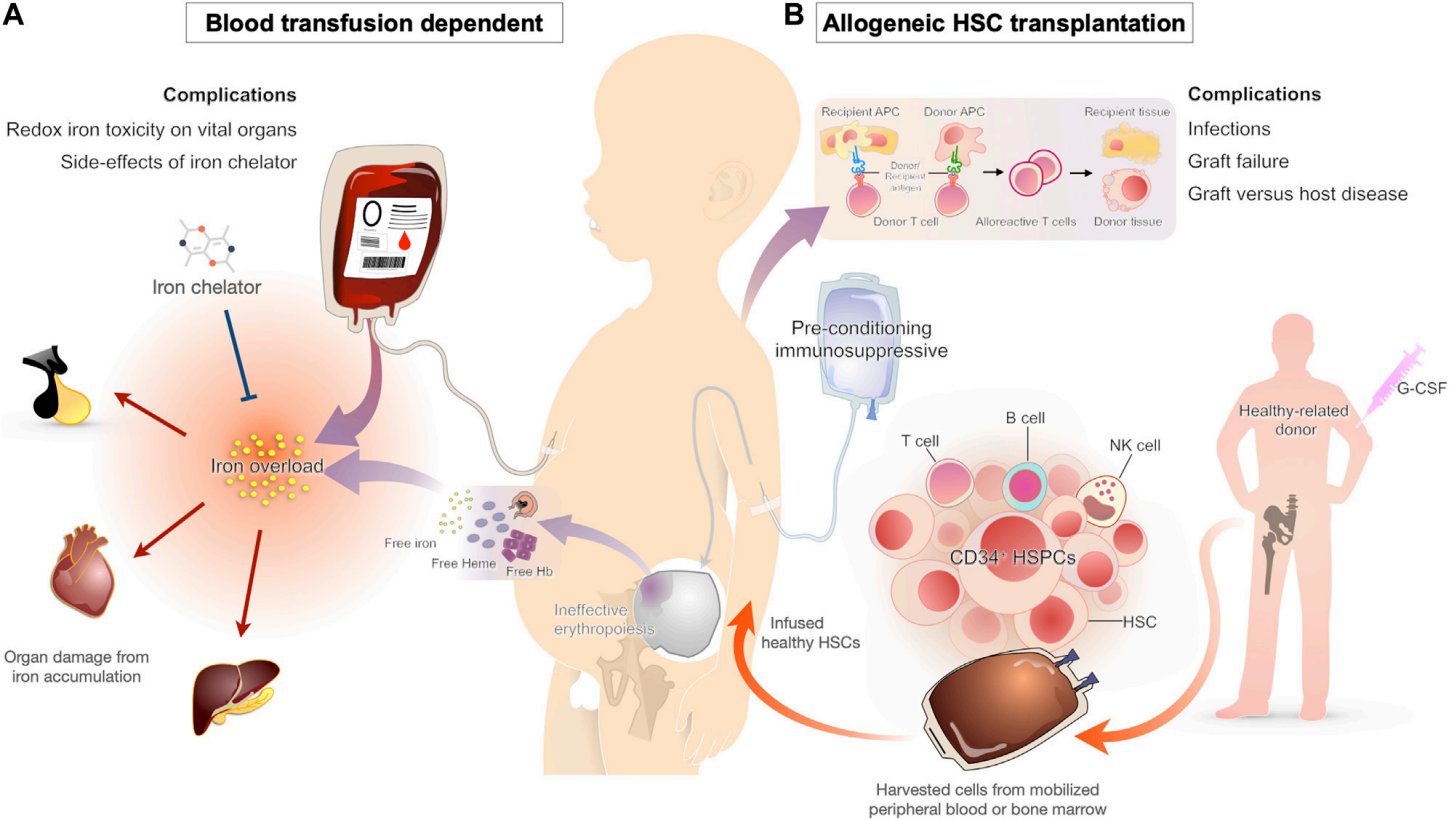
Conventional therapy approaches for β-thalassemia patients and their complications **(A)** Chronic blood transfusion is the standard of care for β-thalassemia patients to maintain adequate hemoglobin levels for effective cardiovascular status. One of the major complications from a blood transfusion is iron overload. As the transfused blood contains iron and the human body lacks a functional iron excretion mechanism, all individuals treated with chronic transfusion develop iron overload, which leads to organ damage from oxidative injury. Iron chelators are typically started early in transfusion-dependent β-thalassemia to prevent the complications of transfusional iron overload **(B)** HSC transplantation has been used for decades as the curative approach for β-thalassemia patients. The HSC replacement substitutes ineffective erythropoietic stem cells with effective cells from a healthy donor. Even with the improvement of transplant technologies during the last decade, severe complications such as infections, graft versus host disease, and graft failure are occasionally seen.

Generally, the treatment approaches in thalassemia include anemia correction, suppression of ineffective erythropoiesis, and iron management. The available treatments for β-thalassemia consist of several therapeutic modalities ranging from conventional treatments such as blood transfusion combined with iron chelation therapy, splenectomy, and hematopoietic stem cell (HSC) transplantation (Figure 1) to recently approved novel treatments such as luspatercept, an activin receptor fusion protein that improves erythropoiesis, and cell and gene therapy (Taher et al., 2018; Motta et al., 2020).

## Current and Future Therapies for Transfusion-dependent β-thalassemia

### Transfusion Therapy for β-thalassemia

Transfusion therapy is considered a mainstay treatment in thalassemia patients (Figure 1). The blood transfusion serves to provide normal RBCs and suppress the ineffective erythroid proliferation, which prevents the downstream pathophysiological consequences (Cazzola et al., 1995). In the absence of blood transfusions, most patients with β-thalassemia major die within the first 5 years of birth (Rachmilewitz and Giardina, 2011). For this reason, regular blood transfusions are recommended in early childhood as soon as the clinical manifestations develop. As there is no controlled mechanism for iron excretion in the human body, chelation therapy is typically needed within 1 year after starting the transfusion regimen (Franchini et al., 2017; Shah et al., 2019).

With the advancements in transfusion and iron chelation therapy in the recent decades, the life expectancy of transfusion-dependent β-thalassemia patients has improved significantly in high-income countries (Borgna-Pignatti et al., 2004; Ladis et al., 2011). Nonetheless, despite the improvements in survival, the quality of life in thalassemia patients undergoing conventional non-curative management remains limited compared with curative therapies (Caocci et al., 2016; Badawy et al., 2021). As regular transfusion regimens require missing school or work for 1 day every 3–4 weeks, in addition to the risk of complications from transfusions and iron chelation therapies, the non-curative treatments may greatly affect patient’s daily activities.

### Cell and Gene Therapy for the Treatment of Transfusion-dependent β-thalassemia

#### Hematopoietic Stem Cell Transplantation

Unlike supportive blood transfusions, allogeneic bone marrow transplantation (BMT) or HSC transplantation offers the hope of a definitive cure for patients with transfusion-dependent β-thalassemia. Transplantation with hematopoietic cells from matched related donors has an 80–87% probability of curing young patients (Lucarelli and Gaziev, 2008), suggesting that high resolution human leukocyte antigen (HLA) typing selection is realized. BMT with unrelated donors yields success rates similar to those obtained with the use of matched sibling donors, but with more severe graft versus host disease (GVHD) (La Nasa et al., 2005; Li et al., 2012). Hence, umbilical cord blood (CB) is an alternative cell source for transplantation that requires less stringent HLA matching (Boncimino et al., 2010); however, graft failure due to a reduced number of stem cells during infusion is a major cause of treatment failure (Ruggeri et al., 2011). Overall, graft failure, and GVHD remain significant causes of transplant failure and complications, especially for adult patients (Figure 1). Although stem cell transplantation is an exciting development for young patients with β-thalassemia, precise clinical judgment needs to be made to balance a potential cure with a risk of mortality and morbidity against life-long treatment with blood transfusions and the long-term complication of iron overload (Higgs et al., 2012). BMT drawbacks, such as GVHD and graft failure, may be avoided by the use of autologous HSC transplantation after transduction with therapeutic genes or gene therapy.

#### β-globin Vector Development and Preclinical Evaluation

**FIGURE 2:**
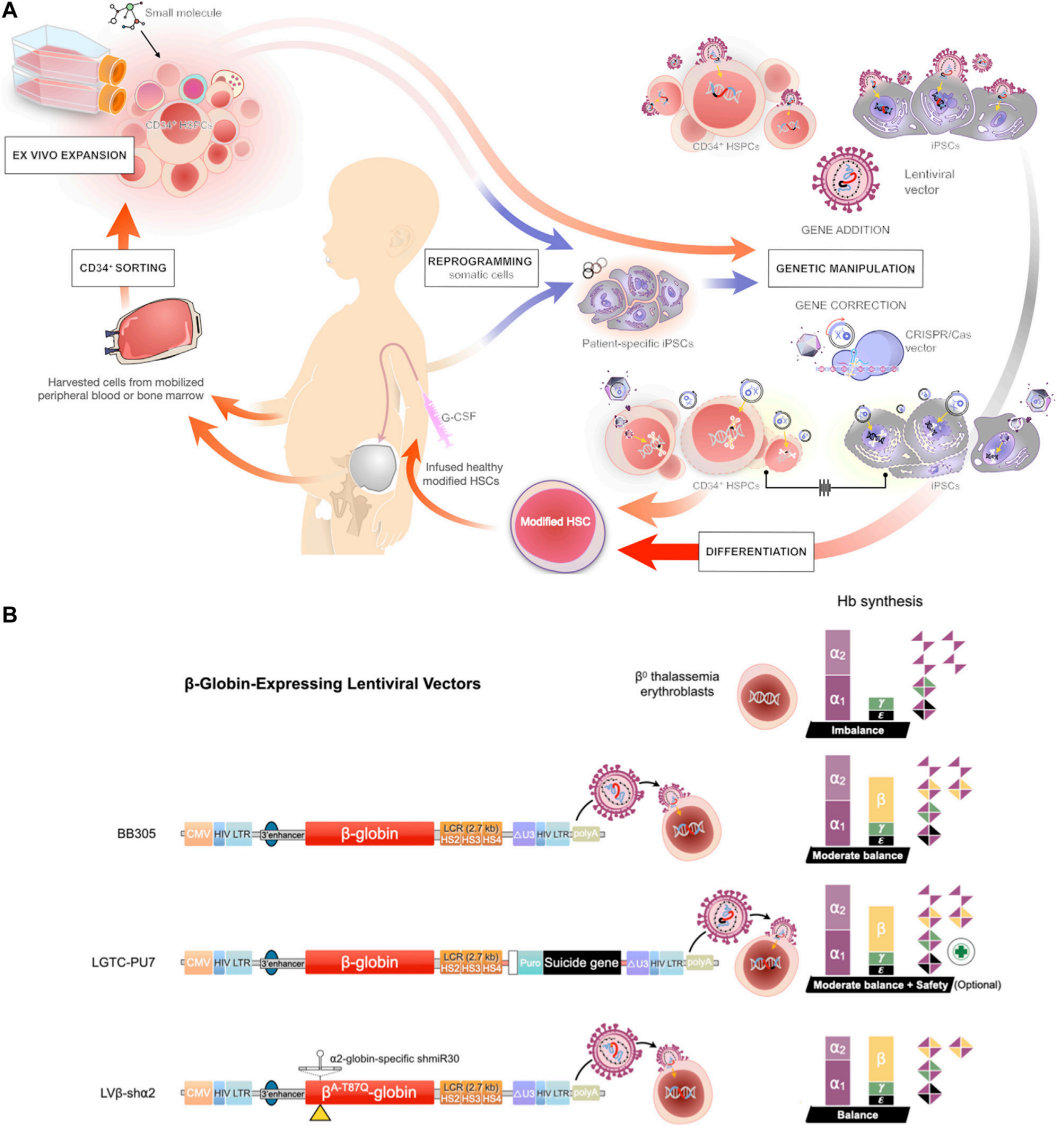
Gene therapy approaches for transfusion-dependent β-thalassemia patients **(A)** Schematic representation of the protocols for gene therapy in β-thalassemia. Induced pluripotent stem cells (iPSCs) are reprogrammed from somatic cells and differentiated into HSCs, or the HSCs are directly harvested from the mobilized peripheral blood or bone marrow of a patient and further manipulated by *ex vivo* maintenance or expanded by co-culture with a cocktail of cytokines and small molecules. The harvested HSCs are subjected to gene transfer (addition) by lentiviral vector or gene editing using CRISPR/Cas9 technology. The engineered HSCs are then applied to replace the inherited defective β-globin gene of a patient and restore function of the erythroid lineage in β-thalassemia **(B)** Prospective of modified lentiviral vector expressing the β-globin gene. The three indicated lentiviral vectors encode the β-globin chain under the control of the human β-globin promoter and hypersensitive sites (HS) of the β-globin locus control region (LCR) (Top) A LentiGlobin™ BB305 vector, the current gene therapy drug product for the treatment of non-β0/β^0^ thalassemia **(Middle)** A modified LentiGlobin™ BB305 vector, LTGC-PU7, encodes the β-globin chain and the puromycin N-acetyltransferase (PAC) cassette with an optional suicide gene **(Bottom)** LentiGlobin™ BB305 is modified to express the miR-30-based short hairpin RNA (shRNA) selectively targeting the α2-globin mRNA, the therapeutic option for β0/β0 thalassemia patients.

Gene therapy refers to a technique involving the introduction of exogenous DNA sequences or therapeutic genes into an appropriate host genome (Figure 2). These therapeutic sequences could have the ability to modify a specific mutation and correct or complement unusual function of the cells in order to overcome a disease (Smith, 2003). Stem cells, particularly HSCs or CD34^+^ cells, are highly attractive target cells for gene therapy because of their ability to reconstitute tissues throughout life. Consequently, most gene therapy approaches for the treatment of hematological disorders focus on targeting the therapeutic gene to repopulating HSCs**.**

β-thalassemia gene therapy is based on the transfer of a human β-globin gene into autologous HSCs, which resolves the absence of compatible donors and eliminates the risk of GVHD and graft failure associated with allogeneic BMT. The aim of autologous HSC gene therapy in β-thalassemia is to provide normal β-globin protein expression. In the past, gamma-retroviral vectors were used to transfer the β-globin gene and its regulatory elements. However, this technique is problematic, as oncoretroviral vectors containing LCR sequences, together with the β-globin regulatory elements, were difficult to produce at high viral vector titers and were very unstable (Novak et al., 1990; Gelinas et al., 1992). Identifying and removing the DNA sequences responsible for vector instability and low titers was intended to improve transduction efficiency (Leboulch et al., 1994; Sadelain et al., 1995); however, condensing the LCR sequence to less than one kilobase (kb) was responsible for high clonal variation in β-globin gene expression both *in vitro* (Sadelain et al., 1995) and *in vivo* (Raftopoulos et al., 1997). Overall, oncoretroviral vectors with these modifications remained sub-optimal at transducing mouse HSCs and had limited capacity for expression of the therapeutic β-globin protein.

Lentiviral vectors, a family of complex retroviruses characterized by stable insertion of their viral genome into the host chromosomes of differentiated lymphocytes and macrophages. The prototype of this virus family is the human immunodeficiency virus-type 1 (HIV-1), a pathogen of the immune system with cytopathic effects, which can be transduced into non-dividing cells arrested at the G1–S boundary of the cell cycle (Naldini et al., 1996a; Naldini et al., 1996b). In addition, lentiviral vectors have the capacity to accept the insertion of large and complex DNA sequences (Kumar et al., 2001) due to the presence of a strong RNA export element (RRE) that binds the RNA-binding viral protein (Cullen, 1998). Side-by-side comparison of lentiviral vectors containing LCR genomic regions of 1 kb versus lentiviral vectors containing longer genomic fractions (3 kb) confirmed that the vector insert size limitation was a major issue for β-globin expression levels (May et al., 2000). Similar lentiviral vectors from two independent groups carrying the β-globin gene (including introns) under the control of the β-globin promoter and LCR elements (2.7–3 kb) enabled efficient transfer and stable integration of the human therapeutic gene in a mouse model of β-thalassemia intermedia (May et al., 2000; Imren et al., 2002). The transduction was sustained in both primary and secondary transplant in immunodeficient mice. Ninety-five percent of the RBCs in all immunodeficient mice who received transplantation contained human β-globin, contributing to one-third of all β-like globin chains. The β-thalassemia phenotype, as assessed by hematological parameters (hemoglobin levels, reticulocytes, and red blood cell counts), was clearly improved. In addition, free α-globin chains were completely cleared from the membranes of RBCs, extramedullary hematopoiesis was ablated, and iron deposits were almost eliminated in liver sections (Imren et al., 2002). In the complete absence of endogenous mouse β-globin gene expression, which is the most severe context of mouse β-thalassemia, the expression level of β-globin per vector copy in transduced RBCs was shown to be approximately half that of the hemizygous endogenous hemoglobin (Hb) production (Rivella et al., 2003). Interestingly, a corrected phenotype in mice with β-thalassemia intermedia was obtained at a transduction rate of 30–50% with cells harboring an average vector copy number (VCN) of 1 (Miccio et al., 2008). In another mouse model of β-thalassemia, a minority proportion of genetically modified HSCs as low as 10–20% of the proportion of normal donor cells resulted in significant improvement of the phenotypes (Persons et al., 2001). These observations are consistent with the preferential survival of normal erythroid cells against a high apoptotic rate of erythroid precursors and RBC hemolysis in β-thalassemia (Centis et al., 2000).

The ability of lentiviral vectors to transduce HSCs from human origin was initially assayed in human umbilical CB cells transplanted into NOD-scid IL2Rγ null immunodeficient mice (NSG mice). Six months after β-globin lentiviral vector transduction and transplantation, around 50% of the human progenitors were genetically modified (Imren et al., 2004), indicating a high transduction efficacy of CB-HSCs by the lentiviral β-globin vectors. The capacity of lentiviral vectors carrying the β-globin gene to correct the β-thalassemia major phenotype was studied in cultured erythroid cells derived from β-thalassemia patients (Centis et al., 2000). Normalized β-globin levels were achieved, leading to effective cell expansion, normal erythroid cell differentiation, and reduction of apoptosis (Puthenveetil et al., 2004; Malik et al., 2005). The gene-corrected human thalassemia CD34^+^ cells were transplanted into NSG mice. Normal levels of human β-globin and effective erythropoiesis were observed in the erythroid progenitor cells derived from human HSCs. Importantly, the expression of β-globin protein was similar to that measured in erythroid colonies derived from normal control subjects (Puthenveetil et al., 2004). Moreover, a number of samples from β-thalassemia patients of different geographic and ethnic origins and from several genotypes (β^0^‐thalassemia homozygous for β^+^ mutations or compound heterozygous for β^0^ and β^+^ mutations) were shown to be transduced *in vitro*. Rescue from apoptosis and correction of ineffective erythropoiesis were potent in most samples (Roselli et al., 2010). As expected, lentiviral β-globin vectors targeted transcriptionally active regions but without bias for cancer-related genes in normal CB stem cells (Imren et al., 2004) as well as in HSCs derived from β-thalassemia patients (Roselli et al., 2010). Preclinical studies further provided the proof of efficacy and safety of those vectors *in vivo* (Ronen et al., 2011; Negre et al., 2015). These studies provided solid preclinical data for the inclusion of patients in clinical trials, with an acceptable risk/benefit ratio.

#### Clinical Trial in β-thalassemia Patients

The first successful use of gene therapy for the treatment of β-thalassemia patients was reported in 2010. In this trial, HSCs were purified and modified to express a β-like globin protein in the erythroid precursors and were then re-infused into patients. Theoretically, the modified HSCs reconstitute the hematopoietic system, thereby producing normal gene-corrected RBCs (Sankaran and Weiss, 2015). The trial, in which lentiviral vector was used to transfer a globin gene into patient-derived HSCs, was planned and announced in 2005 (Bank et al., 2005) and began in 2006 in Paris, France (Cavazzana-Calvo et al., 2010). The first patient in this trial failed to engraft because the purified HSCs had been compromised technically, without relation to the gene therapy vector. The second participant has been carefully followed after the gene transfer procedure. The patient, a male who was 18 years old at the time of treatment, had severe βE/β0-thalassemia and began regular blood transfusion at age 3. His hemoglobin level decreased to as low as 4 g/dl several times, and he did not have a related HLA-matched donor. The patient was conditioned with intravenous busulfan (3.2 mg/ kg per day for 4 days) before transplantation with autologous gene-modified CD34^+^ cells. The transduced CD34^+^ cells contained 0.6 vector copies per cell. One year after transplantation, the patient became transfusion-independent with clear biological and clinical improvement. His hemoglobin level remained stable, between 9 and 10 g/ dl, of which approximately one-third of the total hemoglobin was composed of the therapeutic hemoglobin (β^A−T87Q^). Although correction of the anemia was partial, there was a concurrent decrease in blood reticulocyte and erythroblast counts; however, the hyper-erythroid state remained. The percentage of vector-bearing nucleated blood cells after transplant progressively increased and stabilized to approximately 11%. Chromosomal integration site (IS) analysis of the β^A−T87Q^-globin vector detected a dominant clone in which the vector was inserted in the third intron of the high-mobility group AT-Hook2 (HMGA2) gene. The clone was found in granulocytes, monocytes, and erythroblasts (Cavazzana-Calvo et al., 2010) but not in B and T lymphocytes. The proportion of HMGA2 in peripheral blood remained stable at approximately 2–3% of the circulating nucleated cells (Payen and Leboulch, 2012). A cryptic splice site present in the vector led to the production of an HMAG2 mRNA containing only exons 1, 2, and 3 of the five exons, and the removal of a target site for let-7 microRNA (normally present in exon 5) resulted in increased production of a functional truncated HMGA2 protein. As overexpression of truncated HMGA2 has been involved in benign neoplasia (Cleynen and Van de Ven, 2008), careful and regular follow-up of the patient has been pursued. Currently, the patient is transfusion-independent, with no signs of clonal overgrowth or toxicity.

A recent comprehensive study reported the results of phase 1/2 studies to evaluate the safety and efficacy of gene therapy for β-thalassemia with the use of the LentiGlobin BB305 vector (Thompson et al., 2018), which was modified from a previous LentiGlobin vector (Cavazzana-Calvo et al., 2010). In this study, mobilized autologous CD34^+^ cells were obtained from 22 patents (12–35 years old) and the cells were transduced *ex vivo* with the BB305 vector, which encoded the β^A−T87Q^-globin gene. After the cells were re-infused in the patients, who had undergone myeloblative busulfan conditioning, the efficacy and adverse effects of the vector were observed. The 22 patients were monitored up to 3 years (15–42 months) after transplantation, and no serious adverse events or unexpected safety issues related to the transduced cells have been detected. However, nine of the patients had a severity of the disease that results in microcytic, hypochromic anemia, specifically a β0/β0 genotype. The patients with a β0/β0 genotype showed a reduction in transfusion volume along with decreased annual number of transfusions; indeed, 3 patients stopped RBC transfusion. Interestingly, all of the patients with a βE/β0 genotype, which is the prevalent genotype of β-thalassemia, were able to discontinue transfusions. Of note, treatment-related adverse effects and clonal dominance related to vector integration have not been observed to date (Thompson et al., 2018). In summary, autologous gene therapy with the LentiGlobin BB305 vector transduced in CD34^+^ cells and infused back into patients has been demonstrated as a potential curative treatment in patients with severe β-thalassemia without any adverse events (Biffi, 2018). The clinical data led to the conditional approval of the LentiGlobin BB305 gene therapy vector by the European Commission for transfusion-dependent non-β0/β0 thalassemia in patients 12 years and older. This drug product, owned by bluebird bio, Inc., is the first approved gene therapy for transfusion-dependent β-thalassemia (Harrison, 2019).

In the TIGET-BTHAL study, a phase I/II clinical trial conducted in Italy, 9 patients with β^0^ or severe β^+^ mutations were treated with intrabone autologous genetically modified HSCs using the GLOBE lentiviral vector. Transfusion reduction was observed in adults, and 3 of the 4 children were transfusion independent at the last follow-up. Superior treatment outcomes were observed in younger patients. Higher HSC repopulating capacity and bone marrow function in children could contribute to better clinical benefits (Marktel et al., 2019).

### Current Potentials and Limitations of β-Thalassemia Gene Therapy

Despite their potential for curative outcomes in β-thalassemia, gene therapies contain related concerns, especially the theoretical risks of genotoxicity due to genome manipulation. Post-treatment myelodysplastic syndrome (MDS) was reported 36 months following Lentiglobin infusion in a patient with sickle cell disease. The disease etiology was investigated by multiple molecular- and cytogenetic- assays, which demonstrated no vector integration of CD34^+^ blast cells. Vector-mediated insertional oncogenesis was excluded. In this case, the MDS was likely associated with myeloablative conditioning (Hsieh et al., 2020). Of note, treatment-related adverse effects and clonal dominance related to vector integration and generation of replication have not been observed to date (Cavazzana et al., 2019; Thompson et al., 2019). One case report demonstrated a patient successfully treated with LentiGlobin BB305 drug product and was later diagnosed as wild-type HIV infection. Mostly, the differentiation between wild-type HIV and the lentivirus is difficult to distinguish via polymerase chain reaction (PCR) test as the lentiviral vectors also contain partial HIV-derived gene sequences, which can be false positive for a screening HIV PCR assay in gene therapy patients. In this case, the wild-type HIV infection was confirmed by western blotting and next-generation sequencing. Even though the lentiviral vector is derived from HIV-1, the vector contains a low risk of generating replication-competent virus due to safety modifications in the vector design (Hongeng et al., 2021).

In summary, autologous gene therapy with the lentiviral vector transduced in CD34^+^ cells and infused back into patients has been demonstrated as a potentially curative treatment in patients with severe β-thalassemia. Still, long-term follow-up is critically necessary (Biffi, 2018). In addition to lentiviral vector-based gene therapy, a few ongoing phase I/II clinical trials are currently evaluating the gene-editing approaches using CRISPR/Cas9 and zinc finger nucleases methods, but only in a small number of participants. Indeed, positive preliminary results were observed (Smith et al., 2019; Frangoul et al., 2020), however, more patients and longer follow-up are essential to determine the clinical significance.

### Prospective Approaches for β-thalassemia Treatment

#### Induced Pluripotent Stem Cells: A Promising Prospect for Cell and Gene Therapy

The concept of gene therapy with genetically modified HSCs for the treatment of β-thalassemia has been successfully demonstrated in a new era of medicine. Many researchers have attempted to develop and modify novel strategies to improve future directions of HSC gene therapy, such as manipulation of induced pluripotent stem cells (iPSCs) derived from patient HSCs/somatic cells. iPSCs are autologous somatic cells that are reprogrammed into an embryonic-like stage in vitro. Two key features of human iPSCs are the capacity for self-renewal and pluripotency, which is the ability to differentiate into all cell types (Takahashi and Yamanaka, 2006; Yu et al., 2007), including HSCs (Bisogno et al., 2020; Demirci et al., 2020). Therefore, human iPSCs derived from patients are an attractive cell source for the development of novel strategies for the treatment of hematological disorders (Donada et al., 2020; Kennedy et al., 2012) (Figure 2A). Since the generation of human iPSCs was successfully demonstrated by using different types of cells (e.g., skin or blood) from transfusion-dependent β-thalassemia patients (Papapetrou et al., 2011; Wongkummool et al., 2017). Editing of the endogenous β-globin locus is an attractive strategy; indeed, the β-globin genes mutations can be corrected with various approaches such as CRISPR/Cas9 technology (Song et al., 2015; Niu et al., 2016). However, most of the studies assessed only the potential of hematopoietic differentiation in vitro. In vivo transplantation of HSCs derived from corrected iPSC-derived from β-thalassemia patients into immunodeficient mice has also been demonstrated. They found that the corrected cells could produce hemoglobin, but a low level of hematopoietic cell reconstitution was still observed (Wang et al., 2012; Ou et al., 2016). A recent study used a thalassemic mouse model to mimic thalassemic patients as an implantation model. After transplantation with genetically corrected iPSCs derived from β-thalassemia patients, the corrected cells can differentiate into erythrocytes, nonetheless, an anemia symptom was not effectively recovered (Xian et al., 2020). As genomic instability in the iPSCs, including β-thalassemia patient-derived cells was observed (Ma et al., 2015; Yoshihara et al., 2019). Therefore, a key caution of the iPSCs transplantation is the potential of tumor development, e.g., the induction of p53-mediated DNA damage and cell cycle arrest (Haapaniemi et al., 2018). Estimation of DNA mutation and tumorigenesis of the manipulated-iPSCs are therefore obligatory to test before clinical implication. Moreover, the development of transfusion products from iPSCs would provide autologous RBC transfusion for β-thalassemia patients, despite only allowed a shorter-term treatment. Additionally, in vitro culturing with good manufacturing practice (GMP) platform is always associated with cost raising (Chang et al., 2010; Hirose et al., 2013). Indeed, transfusions of iPSCs-derived RBCs are now representing one of the most promising strategies from iPSC-based therapies (Hansen et al., 2019). However, the expression of many surface antigens of RBCs (blood group system) is still a challenge for clinical application using iPSCs-derived RBCs. To date, although human iPSCs provided a continuous generation of HSCs, the stemness properties of iPSC-derived cells were not completely functional (Tan et al., 2018). Consequently, the engraftment ability of human iPSC-derived HSCs was very low in the animal transplantation model (Li et al., 2017), indicating a long way off to implantation in patients. Thus, it is essential to develop an efficient procedure for human iPSC-derived HSCs to expand and maintain their stemness properties.

#### *Ex vivo* Expansion of HSCs for Cell and Gene Therapy Manipulation

The clinical requirement of HSCs for transplantation is more than 2 million cells/ kg body weight of patients (Hequet, 2015). Thus, to reach therapeutic demand, increasing the number of HSCs by *ex vivo* cell culture would improve transplantation outcomes and permit the use of samples in which the number of HSCs is initially low (Lee et al., 2013). The ability to expand the HSCs and also preserve their functions would be potentially useful in the clinical setting (Psatha et al., 2016). *Ex vivo* expansion using a cocktail of recombinant cytokines has been shown to increase the HSC fraction (Sauvageau et al., 2004). However, some studies have reported that the combination of several cytokines can cause a loss of the ability for self-renewal and can induce HSC differentiation and/or exhaustion (Goff et al., 1998; Ueda et al., 2000). Co-culture of HSCs with stromal cells to simulate the internal hematopoietic niche is another strategy to improve the expansion of HSCs (Jing et al., 2010), but such procedures were also found to produce negative regulators of hematopoiesis (Larsson et al., 2003). A recent study revealed that several small molecules could enhance HSC *ex vivo* expansion by promoting self-renewal, delaying differentiation, increasing homing, and inhibiting apoptosis of HSCs (Zhang and Gao, 2016). For example, a pyrimidoindole derivative, UM171, clearly increased *ex vivo* expansion of long-term HSCs derived from human CB and mobilized peripheral blood (Fares et al., 2014). Therefore, identifying novel small molecules to enhance *ex vivo* HSC self-renewal and preserve HSC pluripotency may be beneficial for HSC expansion, specifically expansion of corrected HSCs (Figure 2A).

#### *Ex vivo* Selection and Optional Suicide Gene of the Genetically Modified HSCs

Some cases of β-thalassemia gene therapy have experienced massive loss of transduced HSCs by contamination with non-transduced cells; thus, the population of genetically modified cells is out of reach for treatment application. Moreover, dominant clonal overexpression of proto-oncogene HMGA2 in erythroid cells was also observed in the first β-thalassemia gene therapy clinical trial (Cavazzana-Calvo et al., 2010). Therefore, various strategies have been considered to improve transduction efficiency to achieve a high level of HSC modification without increasing the concurrent risk of insertional mutagenesis and oncogene activation. We recently demonstrated the possibility of fusing therapeutic genes with selection and suicide genes, including the puromycin *N*-acytyltransferase (PAC) and the herpes simplex virus thymidine kinase (TK) (Figure 2B). We revealed that the puromycin resistant gene allowed optimal *ex vivo* selection of genetically modified HSCs. After selection, transduced HSCs survived and were able to reconstitute human hematopoiesis in immunodeficient mice. Furthermore, the vector was able to express the β-globin gene and produced the suicide protein *in vivo* for elimination of transduced stem cells if necessary (Bhukhai et al., 2018). Thus, expression of PAC and TK cassettes could maintain effective levels of the therapeutic gene, suggesting the procedure for human clinical application with affording the additional safety of conditional suicide gene.

#### Therapeutic Option for β0/β0 Thalassemia

The currently available gene therapy, LentiGlobin BB305, is not approved for the treatment of β0/β0-thalassemia, the severest form of β-thalassemia. Although the treatment could reduce the annual number of RBC transfusions, most of the β0/β0 individuals in clinical trials fail to reach transfusion independence. Moreover, compared with individuals with non-β0/β0 thalassemia, β0/β0 individuals generally require higher VCN of the therapeutic vector to achieve curative levels of Hb, which increases the risk of oncogenicity due to insertional mutagenesis (Thompson et al., 2018; U.S. National Library of Medicine, ; Breda et al., 2012). In many studies, the concept of reducing α-globin synthesis is proposed as an optional modality in gene therapy for β-thalassemia, as the excess α-globin leads to toxic aggregation in RBCs, resulting in immature apoptosis (Mettananda et al., 2016; Sachith et al., 2017). Based on this information, one study has investigated a multiplex lentiviral gene therapy vector with coordinated β-globin expression and α2-globin reduction (LVβ-shα2) modified from LentiGlobin BB305 as an optional strategy for the β0/β0 genotype. LVβ-shα2 demonstrates reduction of α2-globin expression while maintaining expression of the therapeutic β^A−T87Q^-globin gene, which improves α/β-globin balance and decreases cellular damage from unbound α-globin chains in erythroid cells (Figure 2B). Compared with LentiGlobin BB305, LVβ-shα2 also requires a lower VCN of the viral vector for equivalent efficacy, which could improve safety (Nualkaew et al., 2021). This gene therapy approach is promising for curative treatment of the β0/β0 disease, but further studies are required to explore efficacy and reliability in a clinical setting.

## Conclusion

Although the use of lentiviral vectors carrying the β-globin gene has been successfully validated for β-thalassemia treatment, this type of treatment faces challenges involving outcome effectiveness and cost sustainability. Because the long-term consequences of HSC genome editing mechanisms are not completely known, the gene editing strategy may not be considered safer than that of lentiviral-mediated gene transfer. The typical gene addition method remains the most effective therapy to date. Accordingly, challenges the framework of treatment including development of novel directions in gene therapy and fair public access, to get better outcome with effectiveness are urgently required to be available in a large number of β-thalassemia patients.
